# Mindfulness-Based Therapies in the Treatment of Somatization Disorders: A Systematic Review and Meta-Analysis

**DOI:** 10.1371/journal.pone.0071834

**Published:** 2013-08-26

**Authors:** Shaheen E. Lakhan, Kerry L. Schofield

**Affiliations:** Global Neuroscience Initiative Foundation, Los Angeles, California, United States of America; Children's Hospital of Eastern Ontario, Canada

## Abstract

**Background:**

Mindfulness-based therapy (MBT) has been used effectively to treat a variety of physical and psychological disorders, including depression, anxiety, and chronic pain. Recently, several lines of research have explored the potential for mindfulness-therapy in treating somatization disorders, including fibromyalgia, chronic fatigue syndrome, and irritable bowel syndrome.

**Methods:**

Thirteen studies were identified as fulfilling the present criteria of employing randomized controlled trials to determine the efficacy of any form of MBT in treating somatization disorders. A meta-analysis of the effects of mindfulness-based therapy on pain, symptom severity, quality of life, depression, and anxiety was performed to determine the potential of this form of treatment.

**Findings:**

While limited in power, the meta-analysis indicated a small to moderate positive effect of MBT (compared to wait-list or support group controls) in reducing pain (SMD  = −0.21, 95% CI: −0.37, −0.03; p<0.05), symptom severity (SMD  = −0.40, 95% CI: −0.54, −0.26; p<0.001), depression (SMD  = −0.23, 95% CI: −0.40, −0.07, p<0.01), and anxiety (SMD  = −0.20, 95% CI: −0.42, 0.02, p = 0.07) associated with somatization disorders, and improving quality of life (SMD  = 0.39, 95% CI: 0.19, 0.59; p<0.001) in patients with this disorder. Subgroup analyses indicated that the efficacy of MBT was most consistent for irritable bowel syndrome (p<0.001 for pain, symptom severity, and quality of life), and that mindfulness-based stress reduction (MBSR) and mindfulness-based cognitive therapy (MCBT) were more effective than eclectic/unspecified MBT.

**Conclusions:**

Preliminary evidence suggests that MBT may be effective in treating at least some aspects of somatization disorders. Further research is warranted.

## Introduction

Mindfulness-based therapies (MBT) are a clinical application of principles found in Buddhism and other spiritual practices, involving the key element of nonjudgmental acceptance of physical pain or psychological distress, thereby reducing the tendency to ruminate over and catastrophise these experiences [1]–[3]. Interest in applying mindfulness as a therapy developed in Western practice in the late 1970s; early on, the technique of mindfulness-based stress reduction (MBSR) was applied in the treatment of chronic pain [4]. Jon Kabat-Zinn defines mindfulness as “the awareness that emerges through paying attention on purpose, in the present moment, and nonjudgementally to the unfolding of experience moment by moment,” (Kabat-Zinn, 2003, pp.145–146). Mindfulness has been suggested to be effective via four mechanisms: attention regulation, body awareness, emotion regulation, and changes in perspective on the self [5].

Combining elements of MBSR with approaches from cognitive psychology and cognitive-behavioral therapy (CBT) led to the development of mindfulness-based cognitive therapy (MBCT), initially presented as Attentional Control Training [6], and primarily focussed on treating major depression [7], [8]. Over the last several decades, MBT have been applied in treating a variety of physical and psychological disorders. Detailed recent reviews of the use of MBSR, MBCT and other mindfulness and meditation-based approaches in the treatment of depression, anxiety, psychosis, addiction, and physical pain symptoms can be found in Fjorback & Walach [9], Keng et al. [3], and Mars & Abbey [10]. While recognising that mindfulness-based programs can be used in a variety of ways to promote self awareness and personal growth, the primary focus of the present study is on the efficacy of such programs for reducing symptom severity in somatization disorders; the term MBT will therefore be used for present purposes.

### Mindfulness and CBT for Chronic Pain

A number of studies have found psychological therapies, including traditional CBT, to be somewhat effective in the treatment of chronic pain (see [11], [12] for reviews). However, effect sizes for CBT have tended to be moderate [11] while some patients fail to benefit [13]. MBTs have been found to be particularly effective in helping patients to manage chronic pain. A recent systematic review and meta-analysis found that while effect sizes are small to moderate, there is evidence that acceptance and mindfulness play an important role in the acceptance of chronic pain [13].

### Mindfulness and Somatization Disorders

Somatization disorders are characterised by chronic, medically unexplained, treatment-resistant symptoms, combining psychological distress with chronic physical pain or discomfort [14]. Specific somatization disorders include chronic fatigue syndrome (CFS), irritable bowel syndrome (IBS), and fibromyalgia. Studies suggest that CBT has some effectiveness in treating somatization disorders (see [15] for a review) and that cognitive models provide an appropriate theoretical framework for understanding these complex conditions [16]. However, the limitations of CBT noted above apply equally to somatization disorders - for example, only 30% of patients with CFS experience full recovery following conventional CBT [17]. In the last decade in particular, there has been rising interest in exploring MBT in the context of fibromyalgia, CFS and IBS.

### Study Rationale

Mindfulness therapy for somatization disorders is a new approach, for which only a relatively small number of randomized controlled trials currently exist. Previous reviews and meta-analyses have included some of these trials; however, 1) some reviews have grouped MBT in with other complementary “mind-body” approaches, while others have pooled somatization disorders with other chronic pain conditions, 2) those reviews which have included CFS and fibromyalgia have not also included IBS, or mixed presentation somatization disorders, and 3) even in subgroup analyses, only a very small number of randomized controlled trials (RCTs) for the relevant therapy and condition have been included. The present review and meta-analysis, therefore, aims to include all randomized controlled trials from studies which have 1) employed specifically MBT, and 2) involve patients diagnosed specifically with somatization disorder, including IBS and mixed presentations, as well as CFS and fibromyalgia.

## Methods

### Literature Search Strategy

The meta-analysis was performed using Cochrane and PRISMA guidelines [18] (Checklist S1). Studies were identified using PubMed, ScienceDirect, and the Cochrane Library, with the following search criteria: 1) study conducted between the first available year and December 2012; 2) key words: mindfulness, MCBT, MBSR, meditation AND fibromyalgia, chronic fatigue syndrome, CFS, irritable bowel syndrome, IBS, somatization. A manual review of references for each identified study, review, and meta-analysis was also conducted.

### Selection of Studies

Studies were considered acceptable for inclusion in the systematic review if they met the following criteria: 1) MBT was employed (sometimes in conjunction with movement-based therapy, such as yoga or Qigong); 2) patients in the sample had received a diagnosis of fibromyalgia, CFS, IBS, or nonspecified/mixed somatization disorder; and 3) an adult sample was used (18 years or older). Uncontrolled pilot studies, follow-ups, and experimental protocols were accepted for inclusion in the review section of the paper.

For meta-analysis, papers were excluded based on the following criteria: 1) the study was an uncontrolled or partially controlled pilot study (i.e. the method used was not RCT); 2) the study was a case study or included less than six patients in the treatment group (after [19]); 3) insufficient data were available to calculate effect sizes; 4) a pharmaceutical intervention was being trialled in addition to MBT; and 5) movement therapy in the absence of mindfulness was used.

Studies were excluded before review stage as they were either not full-length, peer-reviewed papers (comprising reports of poster or oral conference presentations, letters, comments, editorials) or were themselves reviews of more general MBT or somatization-related research.

### Data Extraction

Numerical data (means, sample sizes, and standard deviations) were extracted from the data for each study for pooled analysis. In order to make scales comparable, mean scores from scales scored in the opposite direction to those from the majority of the studies were inverted.

All studies were considered to have employed psychometrically valid self-report measures. Following Glombiewski et al. [19], the recommendations of The Initiative on Methods, Measurement and Pain Assessment in Clinical Trials (IMMPACT) were used as a guide regarding core outcome domains in clinical trials of pain treatments: pain, physical functioning, emotional functioning, global rating of self-improvement, and adverse events. The most common and comparable outcome measures from the included studies were also assessed, resulting in the following factors: symptom severity, considered to be the primary outcome measure as it was measured across the largest subset of studies, and is the most direct measure of treatment effectiveness; pain; quality of life; depression; and anxiety. Physical functioning was not assessed, as for fibromyalgia this would generally constitute the same measure as symptom severity (the Fibromyalgia Impact Questionnaire (FIQ) is predominantly a score of physical functioning), and, while additional measures of physical function were sometimes presented, these were too diverse across studies to be reliably comparable. Table 1 gives details of the specific measures used in each study.

**Table 1:** Characteristics of included studies for meta-analysis.

Study	Type	N		Female (%)		Intervention	Control	Assessed Outcomes	Findings
		Exp.	Control	Exp.	Control				
**Astin et al, 2003 ** [**27**]	FM	64	64	98.40	100.00	Mindfulness meditation and Qigong	Education/ support	Pain (myalgic score), symptom severity (FIQ), depression (BDI)	Significant improvement in all groups
**Carson et al, 2010 ** [**37**]	FM	25	28	100.00	100.00	Mindfulness meditation and yoga	Wait-list	Pain (myalgic score), symptom severity (FIQ)	Greater improvement in experimental group on symptoms severity
**Goldenberg et al, 1994 ** [**38**]	FM	79	42	90.00	97.00	Stress-reduction CBT with mindfulness	Wait list and not- interested	Pain (VAS); symptom severity (FIQ)	Greater improvement in experimental group on pain; symptom severity showed borderline greater improvement
**Grossman et al, 2007 ** [**39**]	FM	39	13	100.00	100.00	MBSR	Education/support/r elaxation/exercise	Pain (VAS), QoL (Quality of Life Profile for the Chronically Ill), depression (HADS), anxiety (HADS)	Significantly greater improvement in experimental compared to control group on all measures
**Schmidt et al, 2011 ** [**40**]	FM	53	115	100.00	100.00	MBSR	Active control procedure and wait list	Symptom severity (FIQ), QoL (Health Related Quality of Life Scale), depression (CES-D), anxiety (STAI)	Significant improvement in all groups
**Sephton et al, 2007 ** [**30**]	FM	51	39	100.00	100.00	MBSR	Wait list	Depression (BDI)	Significantly greater improvement in experimental compared to control group
**Rimes et al, 2011 ** [**43**]	CFS	16	19	75.00	89.00	MBCT	Wait list	Symptom severity (Chalder Fatigue Scale), depression (HADS), anxiety (HADS)	Significantly greater improvement in experimental compared to control group on symptom severity and depression
**Gaylord et al, 2011 ** [**53**]	IBS	36	39	100.00	100.00	Mindfulness-based pain and stress management	Support group	Pain (abdominal pain subscale of IBS-SS), symptom severity (IBS-SS total score), QoL (IBS-QoL), depression (BSI-18), anxiety (BSI-18)	Significantly greater improvement in experimental compared to control group on symptom severity; significant improvement on the other scales only at 3 month follow up
**Ljotsson et al, 2010 ** [**51**]	IBS	42	43	83.00	86.00	MBCT	Wait list	Pain (gastrointestinal symptom diary), symptom severity (IBS-GSS); QoL (IBS-QoL), depression (MADRS-S)	Significantly greater improvement in experimental compared to control group on all outcomes
**Zernicke et al, 2012 ** [**56**]	IBS	43	47	90.30	87.20	MBSR	Wait list	Symptom severity (IBS-SS), QoL (IBS-QoL)	Significantly greater improvement in experimental compared to control group on symptom severity; both groups improved over time on QoL
**Fjorback et al, 2012 ** [**57**]	S	59	60	80.00	80.00	Mindfulness therapy	Enhanced treatment as usual	Physical health (SF-36, PCS), Other health-related quality of life measures (SF-36), Illness worry (Whitely-8), Physical symptoms (SCL-90), anxiety and depression (SCL-8)	No difference between experimental and control groups
**Sampalli et al, 2009 ** [**59**]	S	50	26	100.00	100.00	MBSR	Wait list	Symptom severity (somatization subscale of SCL-90-R), depression (SCL-90-R), anxiety (SCL-90-R)	Significant improvement in experimental group but not control group on all measures

BDI  =  Beck Depression Inventory; BSI  =  Brief Symptom Inventory; CES-D  =  Center for Epidemiological Studies depression inventory; CFS  =  chronic fatigue syndrome; Exp.  =  experimental group; FIQ  =  Fibromyalgia Impact Questionnaire; FM  =  fibromyalgia; HADS  =  Hospital Anxiety and Depression Scale; IBS  =  irritable bowel syndrome; IBS-GSS  =  IBS Global Symptom Score; IBS-QoL  =  IBS Quality of Life Instrument; IBS-SS  =  IBS Symptom Severity scale; MADRS  =  Montgomery Asberg Depression Rating Scale; N  =  number of participants; PCS  =  Physical Component Summary; S  =  somatization disorder; SCL-8  =  Symptom Checklist; SCL-90-R  =  Symptom Checklist 90 Revised; SF-36  =  SF-36 health survey, STAI  =  State Trait Anxiety Inventory, Quality of Life subscale; QoL  =  Quality of Life; VAS  =  Visual Analogue Scale.

### Data Analysis

Outcome measures were continuous; the standardised mean difference between experimental and control conditions was calculated for each study. Effect sizes were then calculated for all included studies overall, and for subgroups (diagnosis subgroup, comprising fibromyalgia, IBS, and CFS + general somatization disorders; separate analyses were performed for an additional treatment type subgroup for the primary outcome measure only, comprising MBSR, MCBT, and eclectic or nonspecified mindfulness-based therapy) separately. Hedges' g, a variation of Cohen's d which corrects for small-sample bias [20], and its 95% confidence interval was used to calculate pooled effect sizes reported as standardized mean difference (SMD).

Individual effect sizes were weighted by variance, and pooled across all studies, and for subgroups independently. For each subgroup and all studies overall, heterogeneity was considered high at I^2^≥75%, moderate at I^2^ = 50%, and low at I^2^≤25% (after [21]).

Subgroups were compared by testing for heterogeneity across subgroups rather than across studies. The presence of an overall intervention effect was tested. P-values of <0.05 were considered significant. Calculations were performed using RevMan, version 5.2 [22]. The magnitude of the effect size was interpreted using Cohen's recommendation, with a size of 0.2 considered small, 0.5 moderate, and 0.8 large [19].

Risk for publication bias was assessed using funnel plots.

## Results

**Figure 1:**
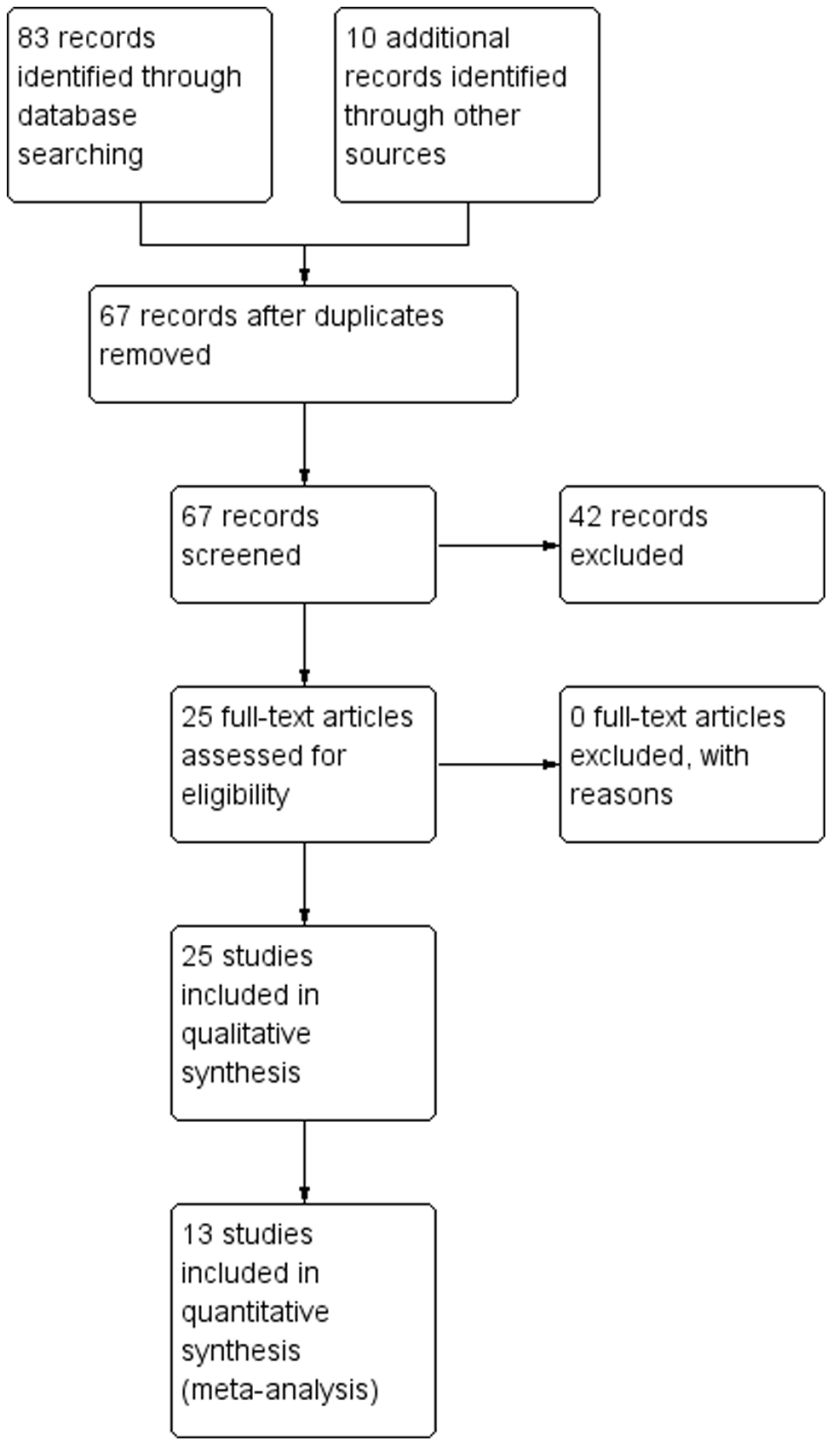
PRISMA diagram showing number of screened, included, and excluded studies.

Of 67 records screened, 25 were included in the qualitative synthesis (systematic review) and 12 in the quantitative synthesis (meta-analysis) (see Figure 1 for flowchart). Those not included in the review were rejected because they did not explicitly include treatments with a mindfulness component, or were reviews or meta-analyses (though some these papers are referenced to provide background information). Table 2 outlines the studies included in the qualitative but not quantitative synthesis and the rationale.

**Table 2:** Characteristics of studies excluded from meta-analysis yet included in systematic review.

Study	Type	Reason for exclusion
**Fors et al, 2002 ** [**25**]	FM	Drug trials, did not explicitly use mindfulness therapy
**Kaplan et al, 1993 ** [**24**]	FM	Uncontrolled pilot study
**Lush et al, 2009 ** [**31**]	FM	Not a clinical trial
**Creamer et al, 2000 ** [**28**]	FM	Did not explicitly use mindfulness therapy
**Weissbecker et al, 2002 ** [**29**]	FM	Same cohort as Sephton et al, 2007 [30]
**Pauzano-Slamm, 2005 ** [**45**]	CFS	Insufficient information available
**Surawy et al, 2005 ** [**44**]	CFS	Uncontrolled trials
**Van Damme et al, 2006 ** [**42**]	CFS	Not a randomized controlled trial, did not explicitly use mindfulness therapy
**Gaylord et al, 2009 ** [**50**]	IBS	Uncontrolled pilot study
**Kearney et al, 2011 ** [**52**]	IBS	Uncontrolled pilot study
**Ljotsson et al, 2010 ** [**55**]	IBS	Uncontrolled pilot study
**Ljottson et al, 2011 ** [**54**]	IBS	Follow-up of previous study with same cohort
**Fjorback et al, 2012 ** [**9**]	S	Alternative measures for existing study with same cohort

CFS  =  chronic fatigue syndrome; FM  =  fibromyalgia; IBS  =  irritable bowel syndrome; S  =  somatization disorder.

### Systematic Review

#### Mindfulness for Fibromyalgia

Fibromyalgia is a chronic pain syndrome, the chief symptom of which is widespread pain with no clear medical cause [23]. Other symptoms associated with the condition include sleep disturbance, depression, and catastrophizing, all of which can potentially be treated with psychological therapies such as CBT or MBSR. Kaplan, Goldenberg, & Galvin-Nadeau [24], in an initial uncontrolled exploration of the effectiveness of MBT for fibromyalgia, found that 51% of a sample of 77 patients showed moderate or marked improvement following the treatment.

A study by Fors, Sexton, & Götestam [25] employed guided imagery techniques, similar in some regards to MBT (training attention on the pain), finding that pleasant, but not attentional imagery reduced pain. This study appears to somewhat contradict the above finding, but did not explicitly use MBT, and included an additional drug trial (see Table 2). A subsequent study of the effects of guided imagery found that the treatment improved functioning and pain-management, but did not reduce pain levels [26]. A randomized controlled study of the effects of MBT on fibromyalgia, by Astin et al. [27], also reported negative findings; however, a similar study by Creamer, Singh, Hochberg, & Berman [28], which did not explicitly include a mindfulness component, found significant improvement in fibromyalgia patients following meditation and Qigong. Related studies by Weissbecker et al. [29] and Sephton et al. [30] found that MBT significantly improved fibromyalgia symptoms, compared to a control group (see Table 1). Following Sephton et al.'s [30] study, an experiment by Lush et al. [31] investigated psychophysiological correlates of the effects of MBT, finding that MBSR was associated with reduced skin conductance level in participants with fibromyalgia.

Previous reviews and meta-analyses have explored the effectiveness of MBT for fibromyalgia. Baranowsky et al. [32] included Astin et al. [27] and Sephton et al.'s [30] studies in a non-meta-analytic review. The two studies together suggested small but significant positive effects in reduction of pain intensity and depression. The same two studies, plus one other [29] were included also in a review by Terhorst, Schneider, Goozdich, Kim, & Stilley [33]. In this case, the Astin et al. study [27] was pooled under movement-based therapies, as it employed a combination of mindfulness therapy and Qigong; Qigong has been shown to have independently reliable positive effects on fibromyalgia symptoms [34]–[36]. The Sephton et al. [30] and Weissbecker et al. [29] studies were pooled under general “mind-body” therapies.

The most closely related meta-analysis to the present study was by Glombiewski et al. [19], who explored psychological treatments for fibromyalgia in general, including MBT as a subset. All psychological treatments were found to be equally effective at improving depression, while CBT and relaxation/biofeedback techniques outperformed other treatments in improving sleep disturbance. CBT outperformed other treatments in reducing pain intensity. However, only two of the twenty-three studies ([27], [30]; see below) used mindfulness therapy; their findings were in opposition to each other.

An additional four recent studies [37]–[40] which employed MBT also demonstrated somewhat conflicting findings; three of which found significantly greater improvement in patients receiving mindfulness therapy compared to controls, while Schmidt et al. [40] reported no difference in improvement between mindfulness and control groups. However, as this study included an “active” control group rather than a waiting list, it is possible that the control group simply received equally effective treatment to mindfulness therapy; this negative finding does therefore not necessarily suggest that mindfulness based therapies are ineffective.

#### Mindfulness for Chronic Fatigue Syndrome

CFS is characterised by severe and debilitating fatigue, lasting for at least six months, usually accompanied by significant impairments in physical, psychological, cognitive, and social functioning [41]. While Van Damme et al. [42] reported evidence for the effectiveness of acceptance in reducing symptom severity and distress, relatively few studies have directly explored the effectiveness of MBT in treating CFS, of which only one was fully controlled, and that was itself a pilot study [43]. However, Surawy, Roberts, & Silver [44] reported three exploratory studies which indicated significant and sustained improvements in subjective levels of fatigue, anxiety, depression, quality of life and physical functioning in patients given mindfulness training. Similarly, Pauzano-Slamm [45] reported cautious support for the efficacy of a mindfulness-based program in improving anxiety, overall psychological distress, fatigue, and overall level of activity.

#### Mindfulness for Irritable Bowel Syndrome

IBS is a common disorder, estimated to affect 5–11% of the population [46]. Studies indicate that psychological treatments such as CBT, psychodynamic therapy, and hypnotherapy can be somewhat effective in ameliorating IBS symptoms [47]. However, improvement is moderate and inconsistent; IBS remains for most patients a chronic condition, and therefore mindfulness approaches, with their focus on acceptance and global change, has potential to be particularly efficacious [48]. This is indirectly supported by findings that pain catastrophizing accounts for 46% of the variance in suffering in IBS patients [49].

It is not surprising, therefore, that there is developing interest in the use of MBT to treat IBS. Gaylord et al. [50] devised a protocol for undertaking randomized control trials to determine efficacy, while Ljótsson, Andréewitch, et al. [51] and Kearney, McDermott, Martinez, & Simpson [52] conducted uncontrolled pilot studies, finding clinically significant and sustained improvements in outcome measures. Three studies [53]–[56] subsequently employed randomized controlled trials, all finding evidence for sustained improvement in IBS symptoms following MBT.

#### Mindfulness for Somatization Disorder (Nonspecified)

In addition to the above, two studies [57]–[59] were conducted as controlled trials of MBT for general somatization disorder (i.e. patients with fibromyalgia, CFS, IBS and other related conditions were pooled into one treatment group). Both demonstrated significant and sustained improvement in clinical outcome measures following MBT.

### Characteristics of Included Studies for Meta-Analysis

For meta-analysis, six studies included patients with fibromyalgia, three with IBS, one with CFS, and two with general or nonspecific somatization disorder (see figure 1). Table 1 gives the methods and findings for each of the included studies, organized by condition (FM, CFS, IBS, somatization), then alphabetically (by authors' last name).

In all studies, participants were predominantly female, and in more than half the studies, the entire sample was female. This may be taken to imply selection or sampling bias; however, it also reflects the greater proportion of women, compared to men, who are diagnosed with somatization disorders ([60] for CFS; [61] for IBS; [62] for fibromyalgia).

### Publication Bias

**Figure 2:**
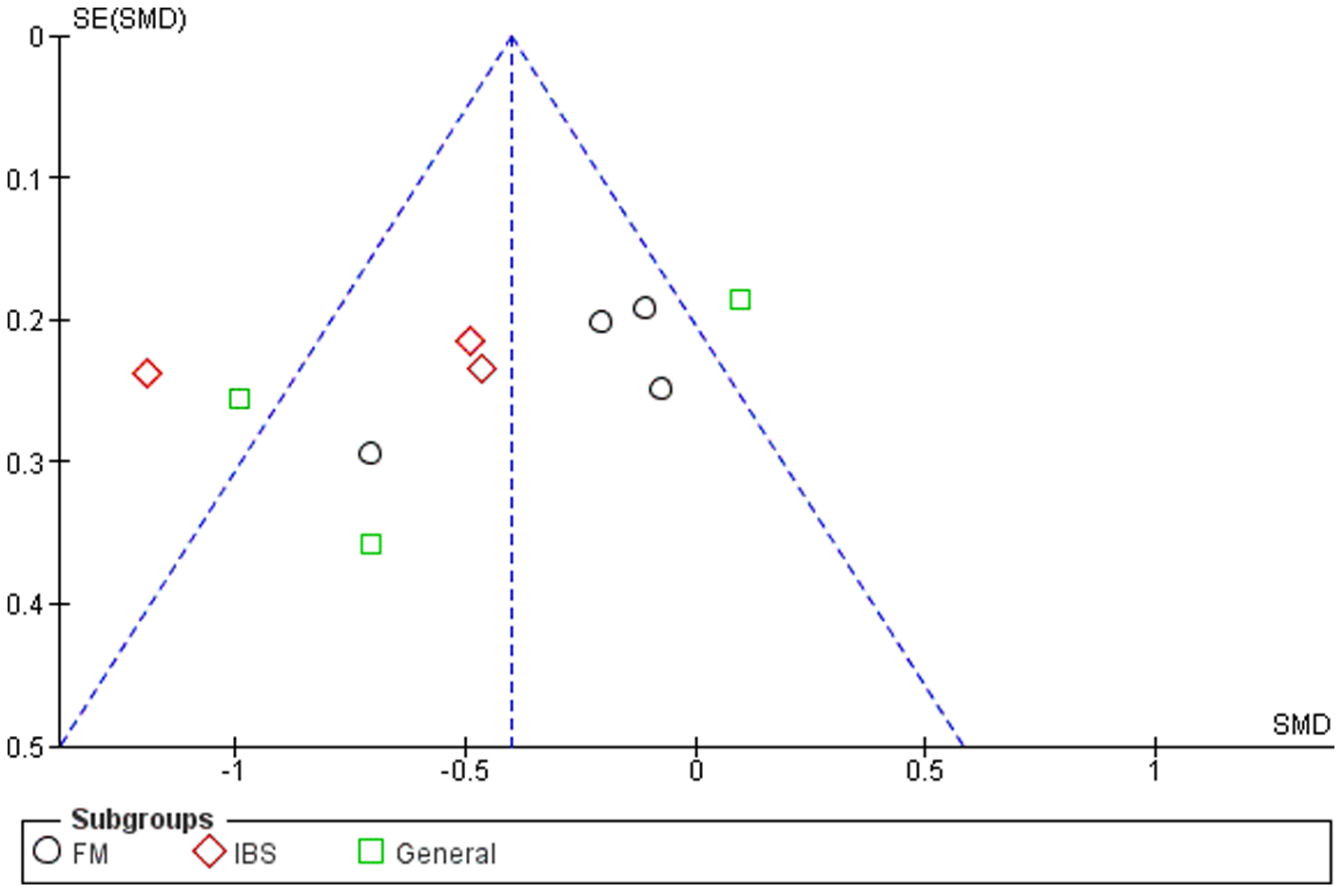
Funnel plot for pain.

**Figure 3:**
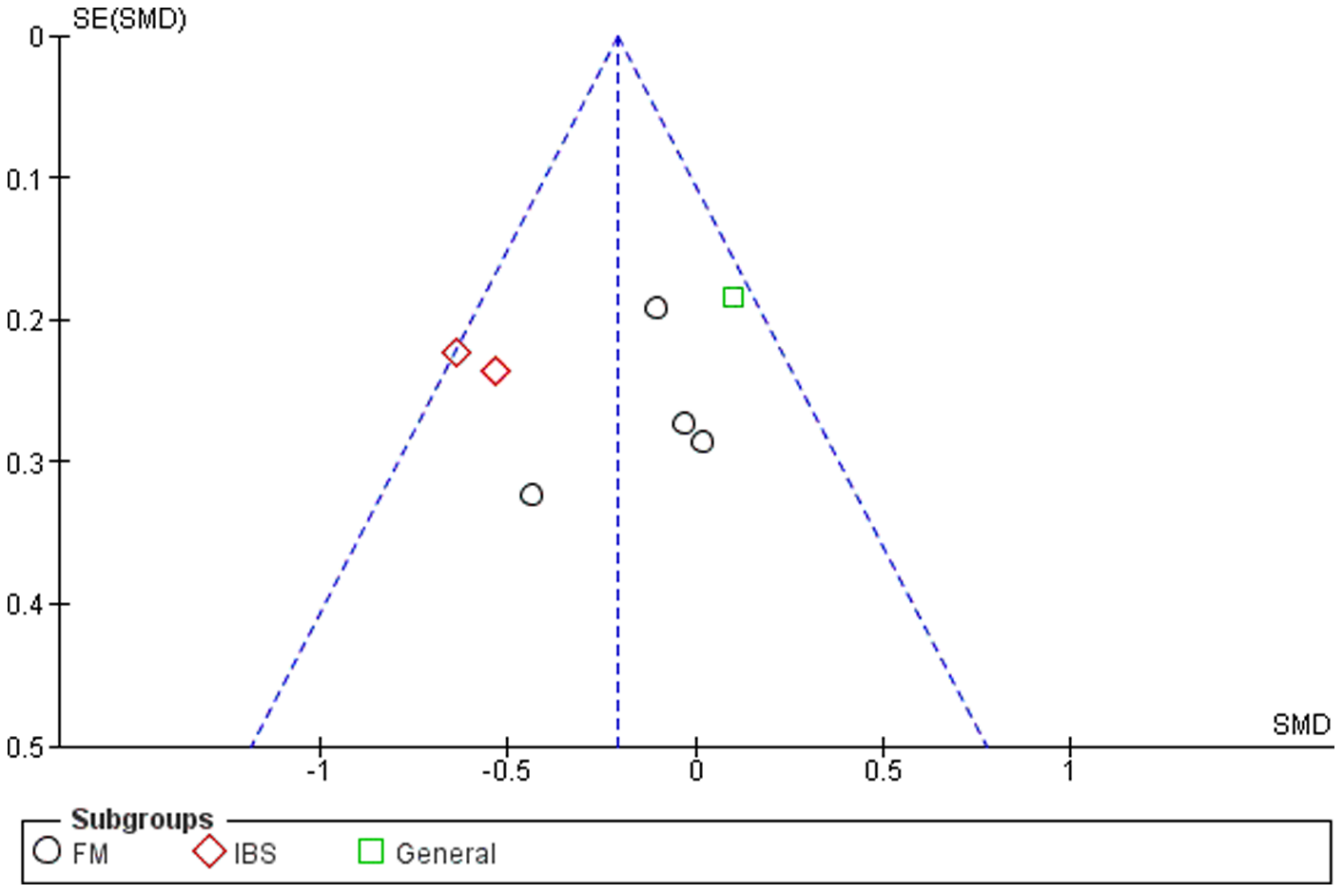
Funnel plot for symptom severity.

**Figure 4:**
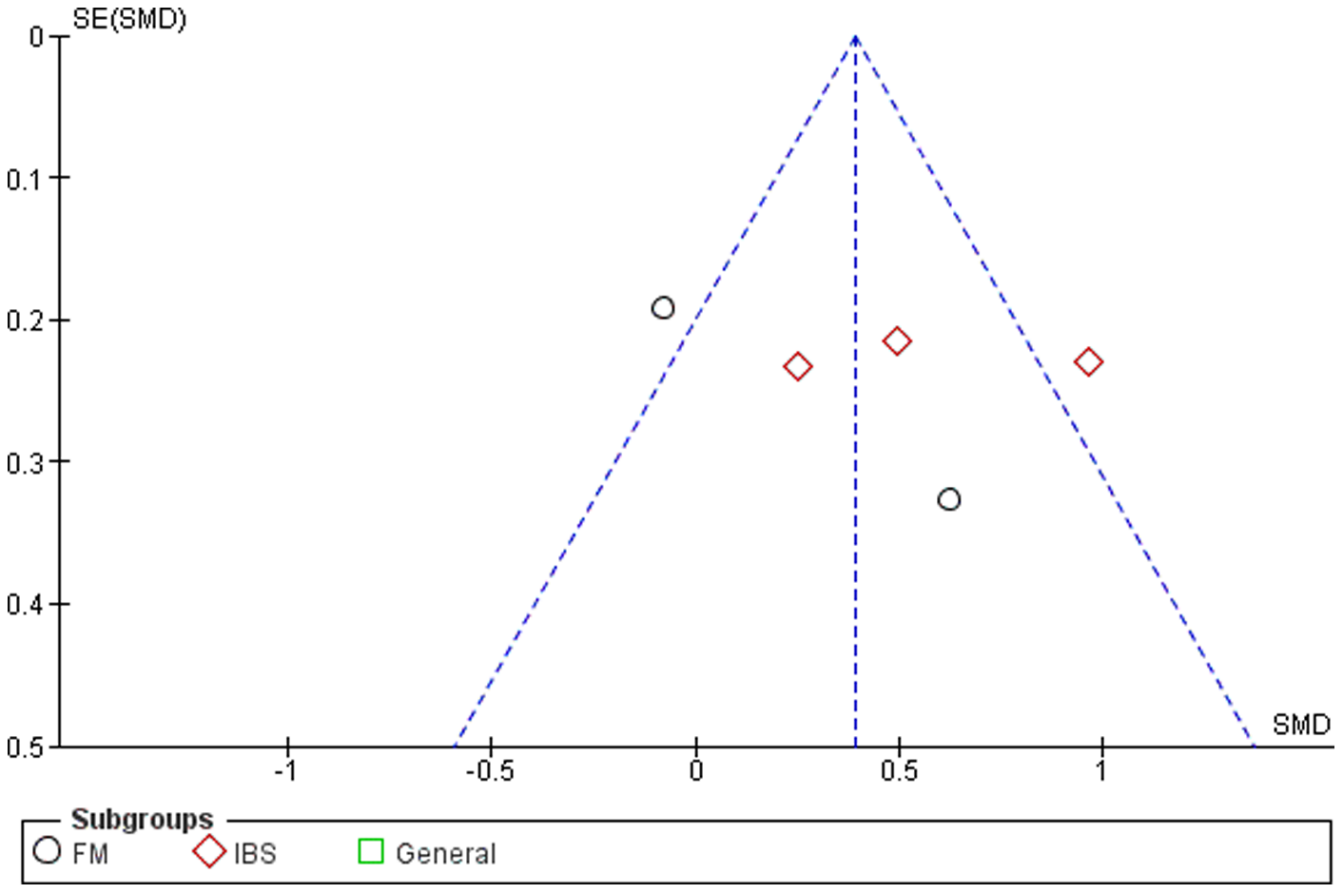
Funnel plot for quality of life.

**Figure 5:**
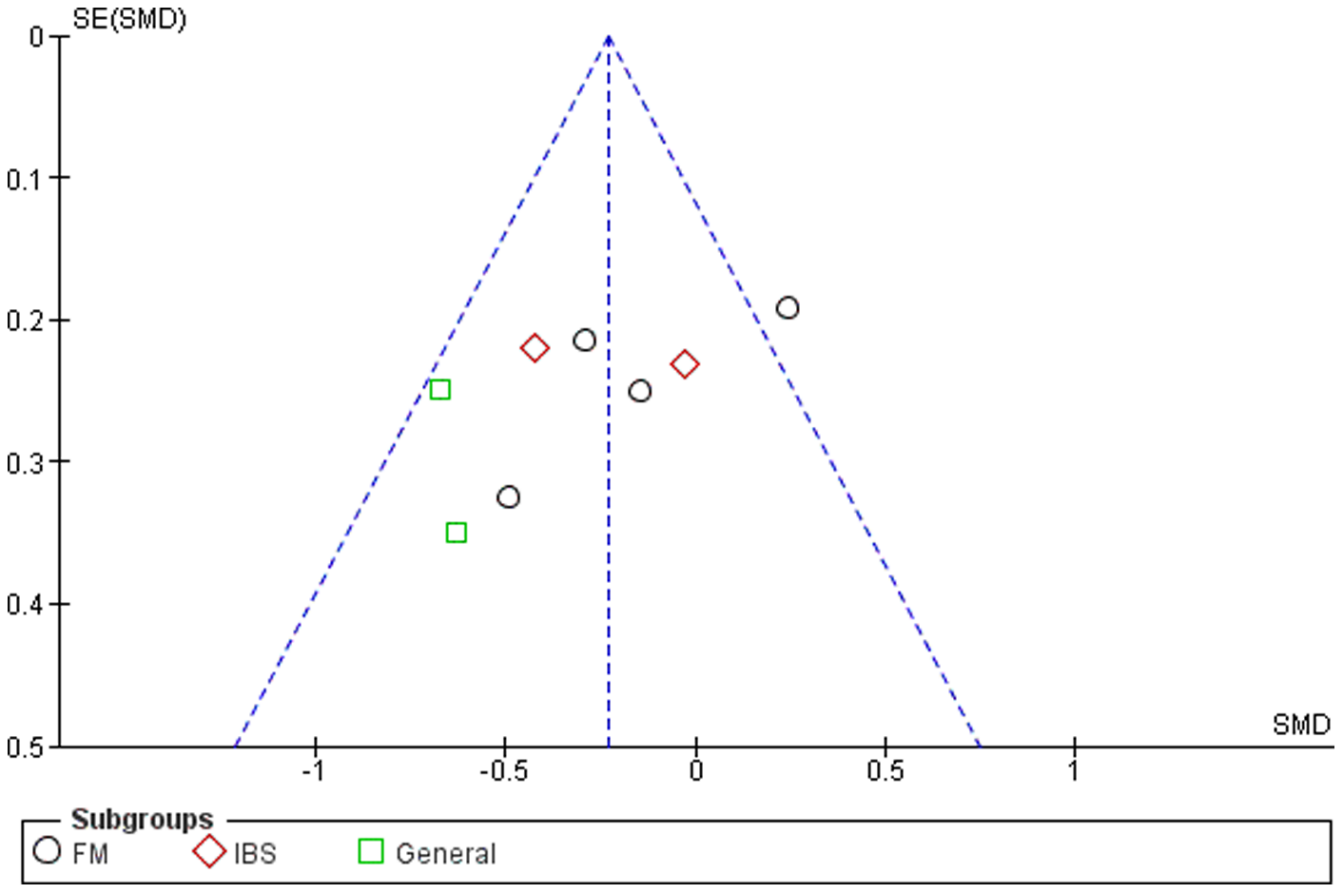
Funnel plot for depression.

**Figure 6:**
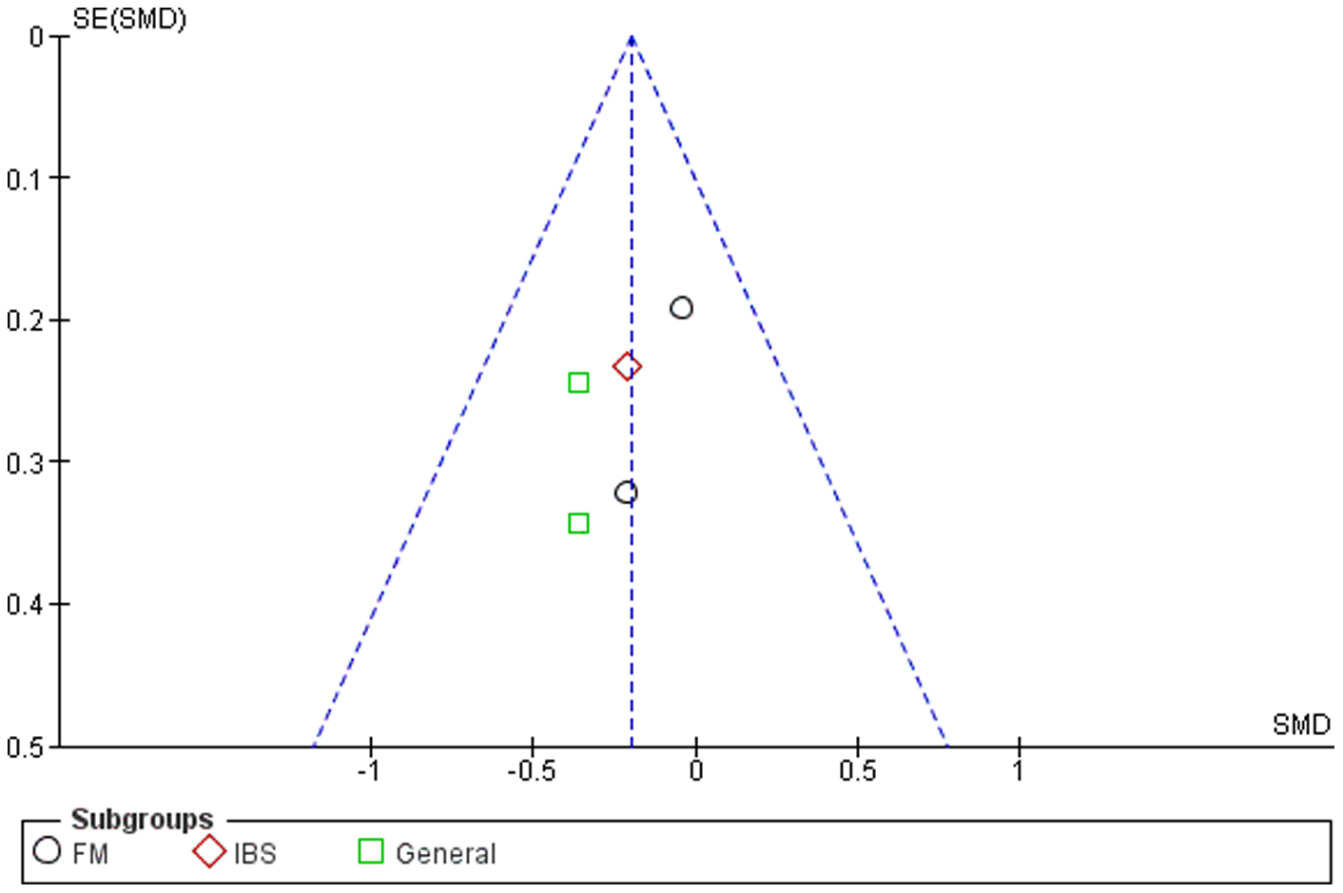
Funnel plot for anxiety.

Although the funnel plots for several outcomes were not fully symmetrical (see Figures 2–6), publication bias could not be concluded. This was partly due to the difficulty of interpreting funnel plots for such a small subset of studies, but also due to the presence of alternative explanatory factors: considerable heterogeneity (see below) and small effect sizes.

### Mindfulness-Based Therapy for Symptom Severity (primary outcome measure)

Ten of the twelve studies included comparable measures of symptom severity. The SMD for symptom severity was small to moderate (−0.40, 95% CI: −0.54, −0.26), significantly in favor of the experimental group (Z = 5.52; p<0.001) (see Figure 3). Heterogeneity was moderately high (I^2^ = 71%).

**Figure 7:**
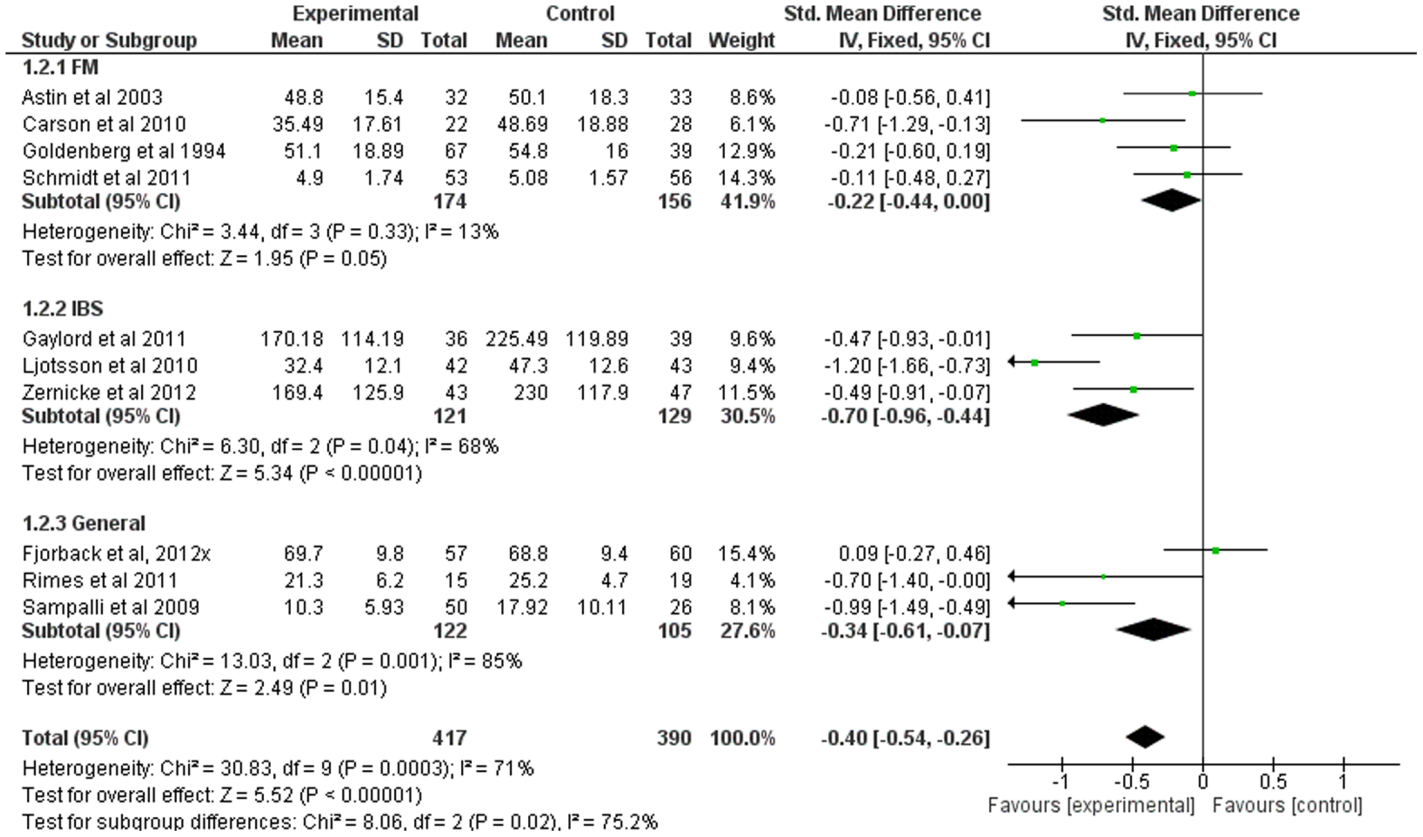
Forest plot showing the effect of mindfulness therapy on the symptom severity outcome measure. Standardised mean difference between experimental and control group indicates that the mindfulness-based therapy group showed significantly more improvement than the control group, overall.

Subgroup analyses indicated considerable heterogeneity between subgroups (I^2^ = 75%; heterogeneity was low or absent for fibromyalgia, moderate to high for the others). SMD was significantly in favor of the experimental group for fibromyalgia (p = 0.05), IBS (p<0.001), and general somatization (p = 0.01) (see Figure 7).

**Figure 8:**
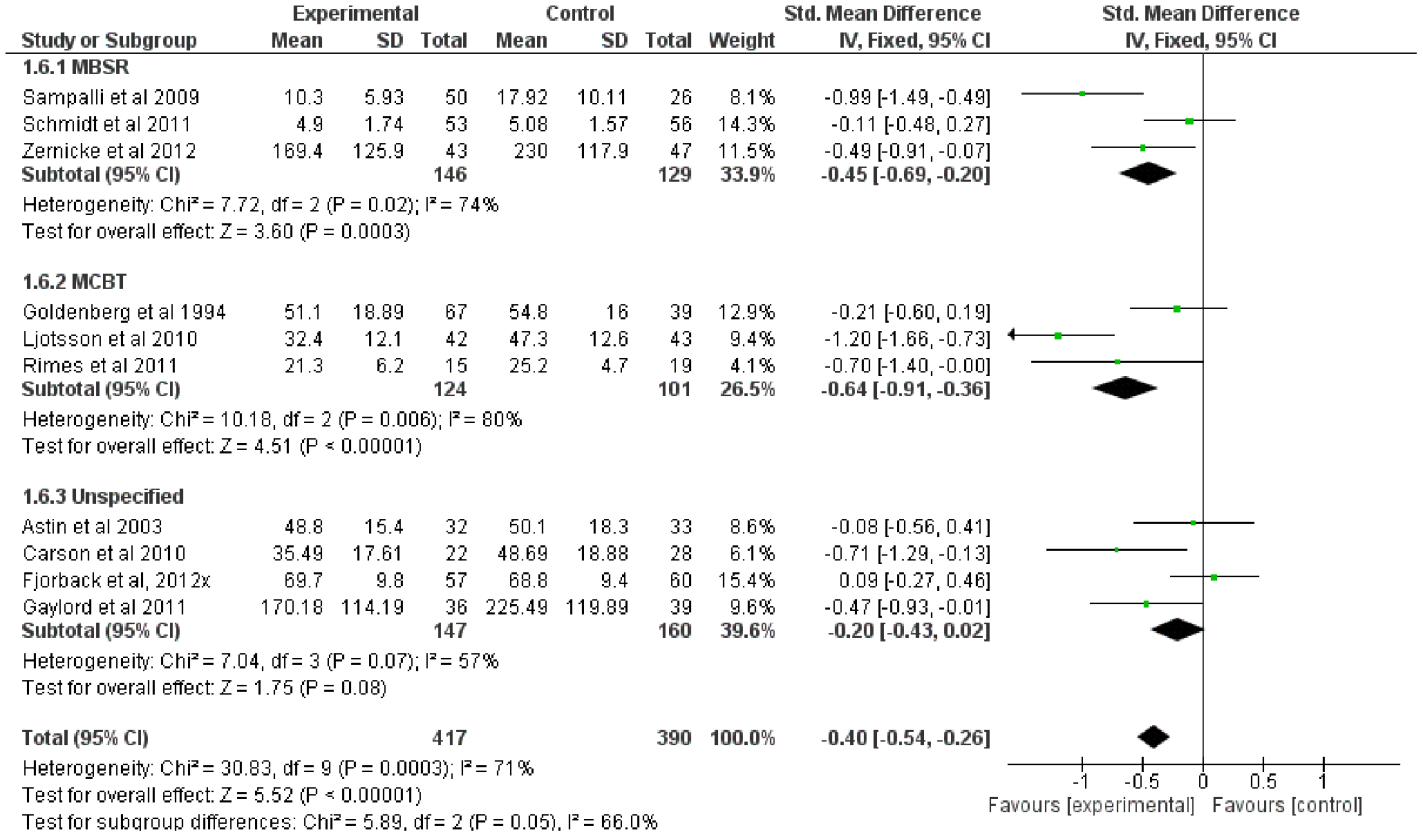
Forest plot showing the effect of type of mindfulness therapy on the symptom severity outcome measure. Standardised mean difference between experimental and control group indicates that the MBSR and MCBT subgroups showed significantly more improvement than the control group, whereas the eclectic/unspecified subgroup did not.

As symptom severity was the primary outcome measure and included the largest subset of the studies, and as the experimental group was favored for all diagnosis subgroups for this outcome only, an additional subgroup analysis was conducted to compare the effects of different therapy types – MBSR, MCBT, and eclectic or nonspecific mindfulness-based therapy. Heterogeneity was moderate to high across subgroups (I^2^ = 66%). SMD was significantly in favor of the experimental group for MBSR and MCBT (both p<0.001), but nonsignificant for the eclectic/unspecified subgroup (p = 0.08) (see Figure 8).

### Mindfulness-Based Therapy for Pain

Seven of the twelve studies included comparable measures of pain severity. The SMD for pain was small (−0.21, 95% CI: −0.37, −0.03), but significantly in favor of the experimental group (Z = 2.31; p<0.05) (see Figure 2). Heterogeneity was moderate (I^2^ = 42%).

**Figure 9:**
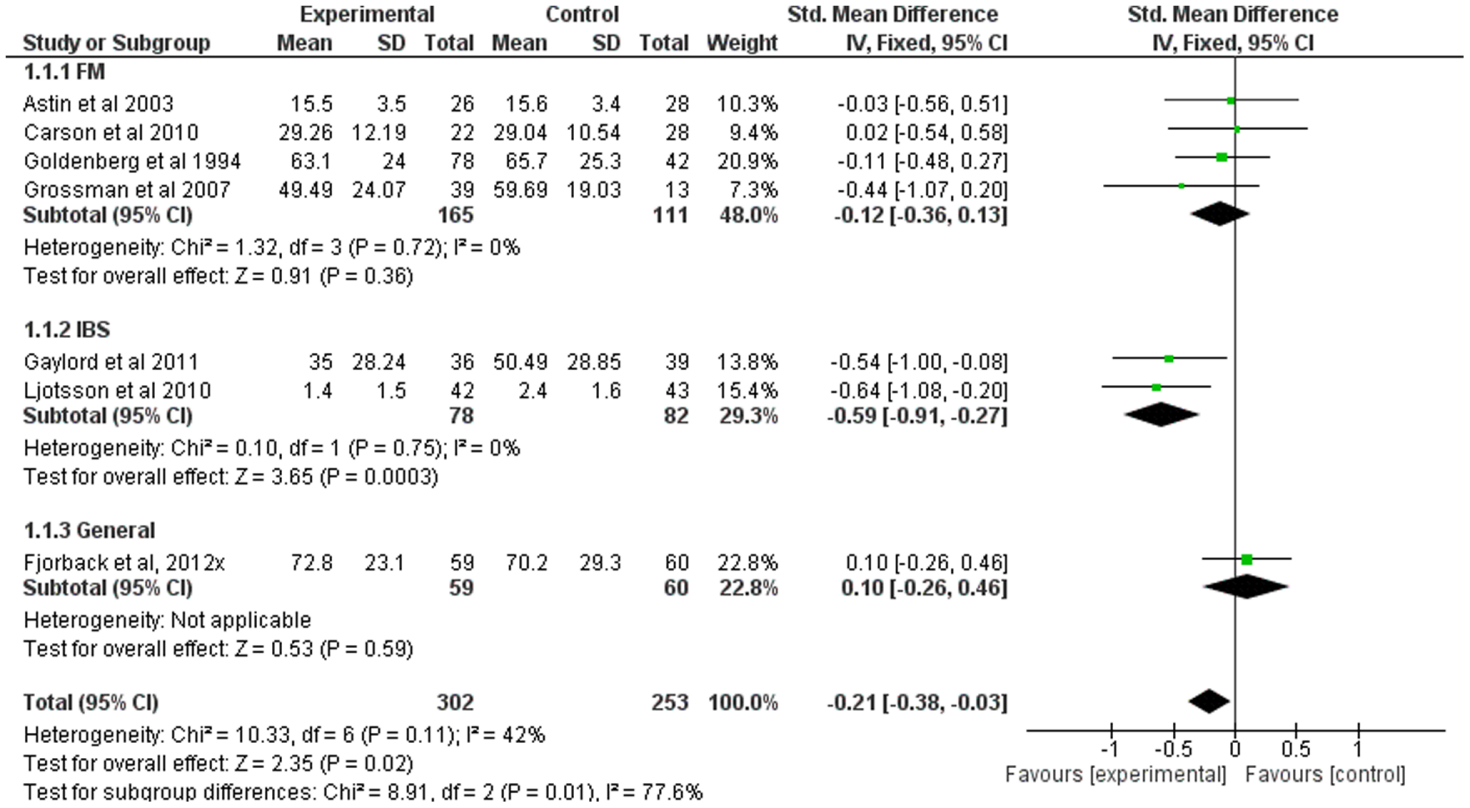
Forest plot showing the effect of mindfulness therapy on the pain outcome measure. Standardised mean difference between experimental and control group indicates that the mindfulness-based therapy group showed significantly more improvement than the control group, in only IBS.

Subgroup analyses indicated considerable heterogeneity between subgroups (I^2^ = 78%;). Heterogeneity was low or absent *within* all subgroups. SMD was significantly in favor of the experimental group for the IBS subgroup (p<0.001) only (see Figure 9).

### Mindfulness-Based Therapy for Quality of Life

Five of the twelve studies included comparable measures of quality of life. The SMD for quality of life was small to moderate (0.39, 95% CI: 0.19, 0.59), significantly in favor of the experimental group (Z = 3.79; p<0.001) (see Figure 4). Heterogeneity was high (I^2^ = 71%).

**Figure 10:**
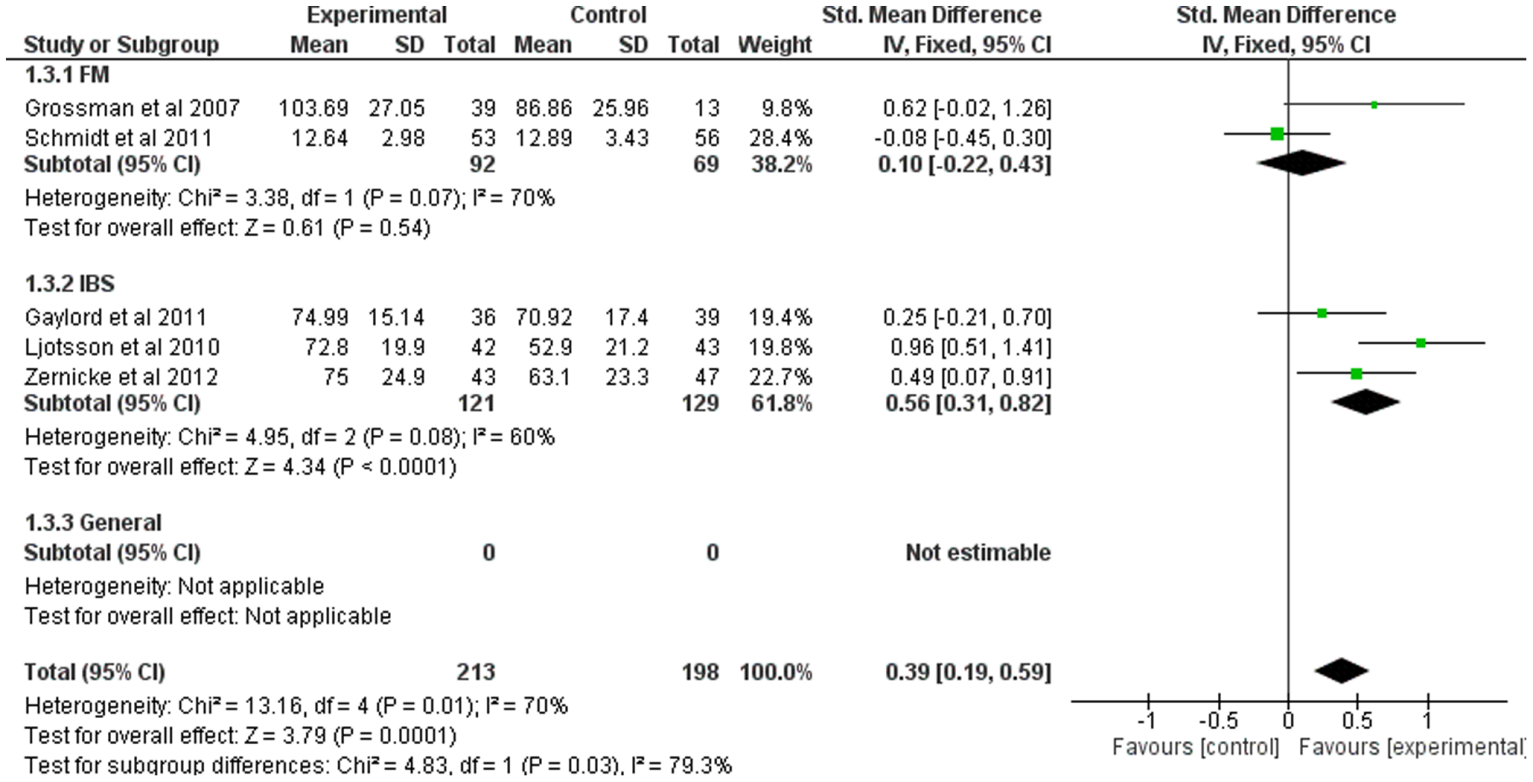
Forest plot showing the effect of mindfulness therapy on the quality of life outcome measure. Standardised mean difference between experimental and control group indicates that the mindfulness-based therapy group showed significantly more improvement than the control group, in only IBS.

Subgroup analyses indicated considerable heterogeneity between subgroups (I^2^ = 79%; heterogeneity was high within all subgroups). SMD was significantly in favor of the experimental group for the IBS subgroup (p<0.001) only (see Figure 10).

### Mindfulness-Based Therapy for Depression

Eight of the twelve studies included comparable measures of depression. The SMD for depression was small overall (−0.23, 95% CI: −0.40, −0.07), but significant in favor of the experimental group (Z = 2.75; p<0.01) (see Figure 5). Heterogeneity was moderate (I^2^ = 67%).

**Figure 11:**
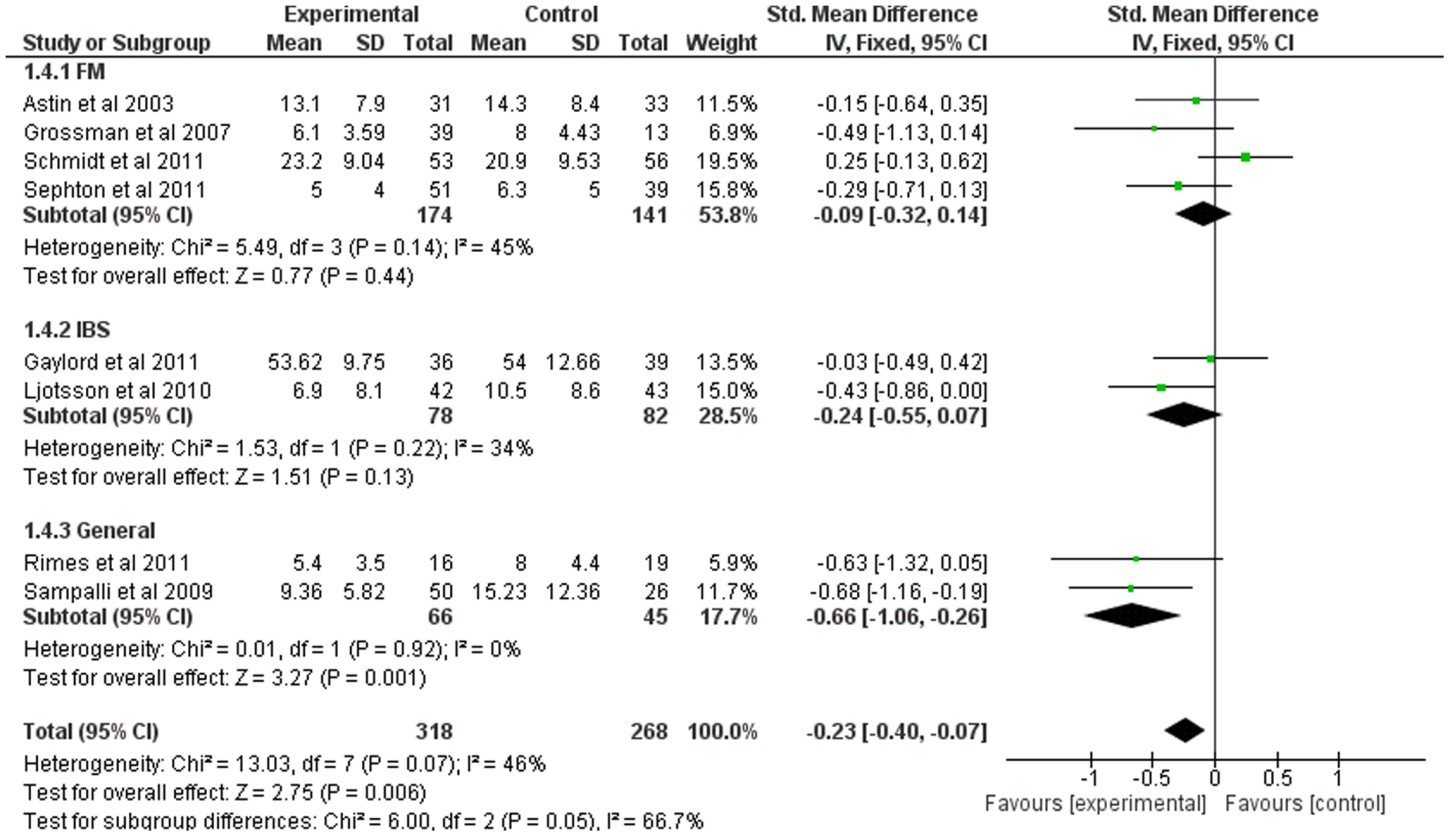
Forest plot showing the effect of mindfulness therapy on the depression outcome measure. Standardised mean difference between experimental and control group indicates that the mindfulness-based therapy group showed significantly more improvement than the control group, in only general somatization.

Subgroup analyses indicated considerable heterogeneity between subgroups (I^2^ = 67%; heterogeneity was moderate for fibromyalgia and IBS, absent for the general somatisation group). SMD was significantly in favor of the experimental group for the general somatization subgroup (p = 0.01) only (see Figure 11).

### Mindfulness-Based Therapy for Anxiety

Five of the twelve studies included comparable measures of anxiety. The SMD for anxiety was small overall (−0.20, 95% CI: −0.42, 0.02), showing a borderline significant trend in favor of the experimental group (Z = 1.82; p = 0.07) (see Figure 6). Heterogeneity was absent (I^2^ = 0).

**Figure 12:**
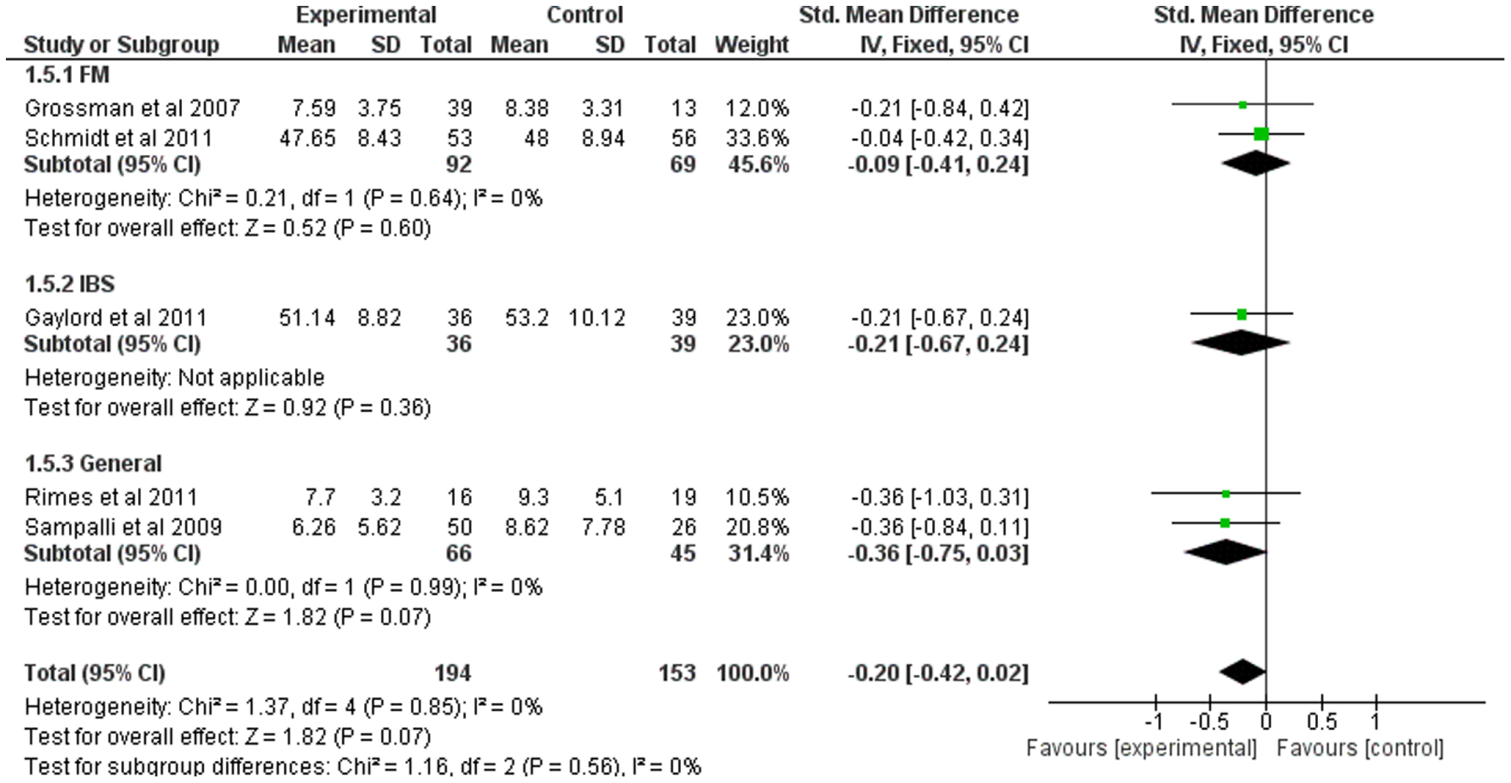
Forest plot showing the effect of mindfulness therapy on the anxiety outcome measure. Standardised mean difference between experimental and control group indicates that the mindfulness-based therapy group had no statistically significant differemce than the control group.

Subgroup analyses indicated no heterogeneity between or within subgroups. SMD was not significantly different in the experimental group versus control (FM p = 0.60, IBS 0.36, general somatization p = 0.07) (see Figure 12).

## Discussion

An overall small to moderate effect of MBT (including MBSR, MBCT, and other protocols, including movement-based therapies with mindfulness components) on somatization disorders was found compared to controls (i.e. wait list, education/support). Specifically, MBT appeared efficacious in reducing pain, symptom severity, depression, and anxiety, and improving quality of life.

The effectiveness of MBT appeared to vary according to diagnosis. For patients with fibromyalgia, only the primary outcome measure, symptom severity, appeared to be reduced, with borderline significance. Effects were clearer for IBS – improvement in quality of life, pain, and symptom severity were all found. For the CFS/general somatization group, symptom severity, depression, and anxiety appeared to be improved by MBT.

The only outcome for which improvement was found across all subgroups was symptom severity; this may reflect a less reliable, or diagnosis dependent, efficacy of MBT for the other aspects, or an artefact of the outcome measures used. Subgroup analysis for different therapy types indicated that the more clearly delineated and formal approaches – MBSR and MCBT – were effective.

### Study Limitations

There are several limitations of the present meta-analysis. Firstly, only a small number of studies were ultimately included. However, those that were included met fairly rigorous criteria. All were randomized controlled trials, with a suitable control condition. The small sample size in a number of the studies was compensated for by the use of Hedges' g; nonetheless, for some outcomes, and some subgroups, only an extremely small number studies fulfilled the criteria. Only limited conclusions can therefore be drawn.

A second limitation is that of heterogeneity, which was high in the present study; however, this was to a large extent due to differences between subgroups, which represent an interesting avenue for further study. Heterogeneity within subgroups was also present to some degree, and may reflect differences between measurement scales. This is a particular concern in the case of the quality of life, depression, and anxiety outcome measures: in some cases (for the IBS studies in particular) these factors were measured specifically in the context of the somatization disorder, while in other cases, general quality of life, depression, and anxiety were measured. It is interesting in light of this that MBT appeared to influence quality of life only for IBS patients, as quality of life was exclusively measured as relating to IBS symptoms for these studies.

Heterogeneity between individual studies is not unexpected given the complexity of somatization disorders and the variability of MBT programs – efficacy can be expected to vary due to a variety of factors, such as the ability of the patient to fully participate in the treatment, the severity of their condition, the presence of comorbid disorders, and the effectiveness of the teacher. Clearer diagnostic criteria for somatization disorders, and a greater understanding of their complexity, may be helpful in identifying the factors underlying heterogeneity (see conclusions below).

Another issue is that of bias; in all studies the sample was predominantly, sometimes exclusively, female. Although, as has been argued above, this is to some extent a natural consequence of the differences in prevalence of somatization disorders between males and females, there remains the possibility of selection and/or sampling bias. In addition, while publication bias could not be unambiguously concluded, nor could it be ruled out as at least a partial cause of the asymmetrical funnel plots found for most outcomes.

## Conclusions

Patients with somatization disorder represent a significant financial challenge to the health service, as symptoms are often intractable and long-term care may be required. MBT is a low-cost intervention which has the potential to improve the quality of life of such patients, and reduce the burden on the health service. Some patients may require long-term, multifaceted treatment, of which, it is suggested, MBT is likely to make an effective component. An understanding of the complex neurobiological, psychological, and social causes of somatization disorder, to improve diagnostic accuracy and therefore the capacity to develop a treatment plan tailored to the needs of each patient, is essential.

While the present meta-analysis indicates that MBTs are potentially useful in managing the symptoms of somatization disorder, effect sizes are small, and results remain somewhat ambiguous; the clearest findings are for IBS, for which mindfulness therapies improved pain, symptom severity, and quality of life, though not depression or anxiety. The latter appeared to improve only in the CFS/general group. In addition, formalised approaches – MBSR and MCBT – appear to be more clearly effective in reducing treatment severity than eclectic/nonspecified approaches, which did not achieve significant efficacy as a subgroup in this study. Further randomized controlled studies are recommended, preferably separately by type of diagnosis (fibromyalgia, IBS, CFS) in order to further elucidate subgroup differences, and establish whether MBTs are more clearly effective for particular outcomes in specific subgroups. For the same reason, future reviews and meta-analyses may wish to avoid pooling across subtypes of somatization disorder.

## Supporting Information

Checklist S1 **Preferred Reporting Items for Systematic Reviews and Meta-Analyses (PRISMA) checklist.**
(RTF)
