# Supplementary material for: Mindfulness-Based Therapies in the Treatment of Somatization Disorders: A Systematic Review and Meta-Analysis
Source: PLoS One. 2013 Aug 26;8(8):e71834. doi: 10.1371/journal.pone.0071834 (PMC3753315; doi:10.1371/journal.pone.0071834)
Supplement: Checklist S1 — Preferred Reporting Items for Systematic Reviews and Meta-Analyses (PRISMA) checklist. (RTF) [file pone.0071834.s001.rtf]

PRISMA 2009 Checklist

													
	Section/topic			#			Checklist item			Reported			
										on page #			
													
													
												
	TITLE											
													
	Title	1		Identify the report as a systematic review, meta-analysis, or both.	1                                                          			
												
												
	ABSTRACT											
	Structured summary	2			Provide a structured summary including, as applicable: background; objectives; data sources; study eligibility criteria,	1			
							participants, and interventions; study appraisal and synthesis methods; results; limitations; conclusions and				
												
							implications of key findings; systematic review registration number.					
												
												
	INTRODUCTION											
	Rationale	3		Describe the rationale for the review in the context of what is already known.	4			
									
	Objectives	4			Provide an explicit statement of questions being addressed with reference to participants, interventions, comparisons,	3-4			
							outcomes, and study design (PICOS).					
												
												
	METHODS											
	Protocol and registration	5			Indicate if a review protocol exists, if and where it can be accessed (e.g., Web address), and, if available, provide	NA		
							registration information including registration number.			
												
								
	Eligibility criteria	6			Specify study characteristics (e.g., PICOS, length of follow‐]up) and report characteristics (e.g., years considered,	4-5, tables 1 & 2		
							language, publication status) used as criteria for eligibility, giving rationale.					
							
	Information sources	7		Describe all information sources (e.g., databases with dates of coverage, contact with study authors to identify	4, figure 1		
							additional studies) in the search and date last searched.					
									
	Search	8			Present full electronic search strategy for at least one database, including any limits used, such that it could be	4			
							repeated.					
									
	Study selection	9			State the process for selecting studies (i.e., screening, eligibility, included in systematic review, and, if applicable,		5, figure 1		
							included in the meta‐]analysis).					
										
	Data collection process	10			Describe method of data extraction from reports (e.g., piloted forms, independently, in duplicate) and any processes	4-5			
							for obtaining and confirming data from investigators.				
												
									
	Data items	11			List and define all variables for which data were sought (e.g., PICOS, funding sources) and any assumptions and	4-6			
							simplifications made.					
									
	Risk of bias in individual	12			Describe methods used for assessing risk of bias of individual studies (including specification of whether this was	5-6			
	studies					done at the study or outcome level), and how this information is to be used in any data synthesis.					
								
	Summary measures	13		State the principal summary measures (e.g., risk ratio, difference in means).	5-6			
										
	Synthesis of results	14			Describe the methods of handling data and combining results of studies, if done, including measures of consistency	5-6			
							(e.g., I2) for each meta‐]analysis.				
												
							Page 1 of 2					


PRISMA 2009 Checklist

													
	Section/topic			#			Checklist item			Reported			
										on page #			
													
													
										
	Risk of bias across studies	15			Specify any assessment of risk of bias that may affect the cumulative evidence (e.g., publication bias, selective	5-6			
							reporting within studies).					
										
	Additional analyses	16			Describe methods of additional analyses (e.g., sensitivity or subgroup analyses, meta-regression), if done, indicating	5-6			
							which were pre‐]specified.					
												
												
	RESULTS											
	Study selection	17			Give numbers of studies screened, assessed for eligibility, and included in the review, with reasons for exclusions at	Figure 1		
							each stage, ideally with a flow diagram.					
										
	Study characteristics	18			For each study, present characteristics for which data were extracted (e.g., study size, PICOS, follow-up period) and		Table 1		
							provide the citations.				
												
							
	Risk of bias within studies	19		Present data on risk of bias of each study and, if available, any outcome level assessment (see item 12).	NA		
									
	Results of individual studies	20			For all outcomes considered (benefits or harms), present, for each study: (a) simple summary data for each	9-10,			
							intervention group (b) effect estimates and confidence intervals, ideally with a forest plot.	figures 2-6		
									
	Synthesis of results	21			Present results of each meta-analysis done, including confidence intervals and measures of consistency.		7-8, figures		
	Risk of bias across studies	22		Present results of any assessment of risk of bias across studies (see Item 15).	7-11			
					9			
								
	Additional analysis	23		Give results of additional analyses, if done (e.g., sensitivity or subgroup analyses, meta-regression [see Item 16]).	9-10			
												
												
	DISCUSSION											
	Summary of evidence	24			Summarize the main findings including the strength of evidence for each main outcome; consider their relevance to	10-11			
							key groups (e.g., healthcare providers, users, and policy makers).					
									
	Limitations	25			Discuss limitations at study and outcome level (e.g., risk of bias), and at review-level (e.g., incomplete retrieval of	11			
							identified research, reporting bias).					
								
	Conclusions	26		Provide a general interpretation of the results in the context of other evidence, and implications for future research.	11-12			
												
												
	FUNDING											
	Funding	27			Describe sources of funding for the systematic review and other support (e.g., supply of data); role of funders for the		NA			
							systematic review.					
													
													

From: Moher D, Liberati A, Tetzlaff J, Altman DG, The PRISMA Group (2009). Preferred Reporting Items for Systematic Reviews and Meta-Analyses: The PRISMA Statement. PLoS Med 6(6): e1000097. doi:10.1371/journal.pmed1000097
For more information, visit: www.prisma‐]statement.org.

Page 2 of 2
